# Application of digital pathology‐based advanced analytics of tumour microenvironment organisation to predict prognosis and therapeutic response

**DOI:** 10.1002/path.6153

**Published:** 2023-08-08

**Authors:** Xiao Fu, Erik Sahai, Anna Wilkins

**Affiliations:** ^1^ Tumour Cell Biology Laboratory The Francis Crick Institute London UK; ^2^ Biomolecular Modelling Laboratory The Francis Crick Institute London UK; ^3^ Division of Radiotherapy and Imaging Institute of Cancer Research London UK; ^4^ Royal Marsden Hospitals NHS Trust London UK

**Keywords:** advanced analytics, digital pathology, tumour microenvironment, artificial intelligence, biomarker

## Abstract

In recent years, the application of advanced analytics, especially artificial intelligence (AI), to digital H&E images, and other histological image types, has begun to radically change how histological images are used in the clinic. Alongside the recognition that the tumour microenvironment (TME) has a profound impact on tumour phenotype, the technical development of highly multiplexed immunofluorescence platforms has enhanced the biological complexity that can be captured in the TME with high precision. AI has an increasingly powerful role in the recognition and quantitation of image features and the association of such features with clinically important outcomes, as occurs in distinct stages in conventional machine learning. Deep‐learning algorithms are able to elucidate TME patterns inherent in the input data with minimum levels of human intelligence and, hence, have the potential to achieve clinically relevant predictions and discovery of important TME features. Furthermore, the diverse repertoire of deep‐learning algorithms able to interrogate TME patterns extends beyond convolutional neural networks to include attention‐based models, graph neural networks, and multimodal models. To date, AI models have largely been evaluated retrospectively, outside the well‐established rigour of prospective clinical trials, in part because traditional clinical trial methodology may not always be suitable for the assessment of AI technology. However, to enable digital pathology‐based advanced analytics to meaningfully impact clinical care, specific measures of ‘added benefit’ to the current standard of care and validation in a prospective setting are important. This will need to be accompanied by adequate measures of explainability and interpretability. Despite such challenges, the combination of expanding datasets, increased computational power, and the possibility of integration of pre‐clinical experimental insights into model development means there is exciting potential for the future progress of these AI applications. © 2023 The Authors. *The Journal of Pathology* published by John Wiley & Sons Ltd on behalf of The Pathological Society of Great Britain and Ireland.

## Introduction

Visual interpretation of H&E‐stained tissue sections by specialist pathologists using an optical microscope has been the cornerstone of diagnostic oncology since the late 19th century [1]. Over several decades, detailed descriptions of grading systems, such as the Gleason score in prostate cancer or histological grade in breast cancer, have been developed and refined, often involving international working groups of pathologists establishing consensus statements [2, 3, 4, 5, 6]. Similarly, histological variants within tumour types and tumour hallmarks known to represent indolent versus aggressive behaviour have been described and validated, collectively representing a huge wealth of pathological knowledge [7, 8, 9]. Yet in the last 10 years, the application of advanced analytics, especially artificial intelligence (AI), to digital H&E images and other histological image types has begun to radically change how histological images are used in the clinic.

Innovation in advanced analytics, especially AI, has typically occurred outside of oncology but has been rapidly transferred from other disciplines to diagnostic, prognostic or predictive use in oncology. The automated extraction of quantitative data and the elucidation of image patterns not detectable by the human eye are both important areas of additional benefit over microscopic assessment by a pathologist. Recent years have seen a number of AI‐based pathology products obtain regulatory approval, including from the FDA [10, 11, 12]. In a large clinical study across more than 100 institutions, use of a prostate cancer diagnostic AI algorithm was shown to increase the sensitivity of cancer detection and reduce both false positives and false negatives [10]. For a cancer that is very common globally, and for which diagnosis requires meticulous examination of large tissue areas to avoid missing small tumour foci, AI approaches have a number of benefits. In the developed world, this includes a reduction in pathologist workload and fatigue, thus enabling pathologists to focus on the description of tumour foci identified by the AI detection system. In the developing world, the combination of innovative smartphone technology and appropriate validated AI algorithms could help to overcome the substantial clinically important lack in pathology expertise and microscope facilities.

The tumour microenvironment (TME) encompasses the wider cellular and acellular milieu in which tumour cells reside. It primarily consists of innate and adaptive immune cells alongside the tumour vasculature and fibroblasts, which, together with tumour cells, are supported by an extensive ECM. The TME has a pivotal role in shaping tumour phenotype, evolutionary dynamics, and therapy responses [13]. As a result, characterising the spatial relationships between specific cell populations in the TME using digital pathology tools and analytical approaches that are able to capture the TME complexity is a research priority.

This review will present the wide range of digital pathology images to which advanced analytics are currently being applied, as well as the methodology for such analytics, especially those based on machine learning. Finally, we will discuss the feasibility and challenges of widespread incorporation of AI approaches to clinical practice.

### Image types for advanced analytics

**Figure 1:**
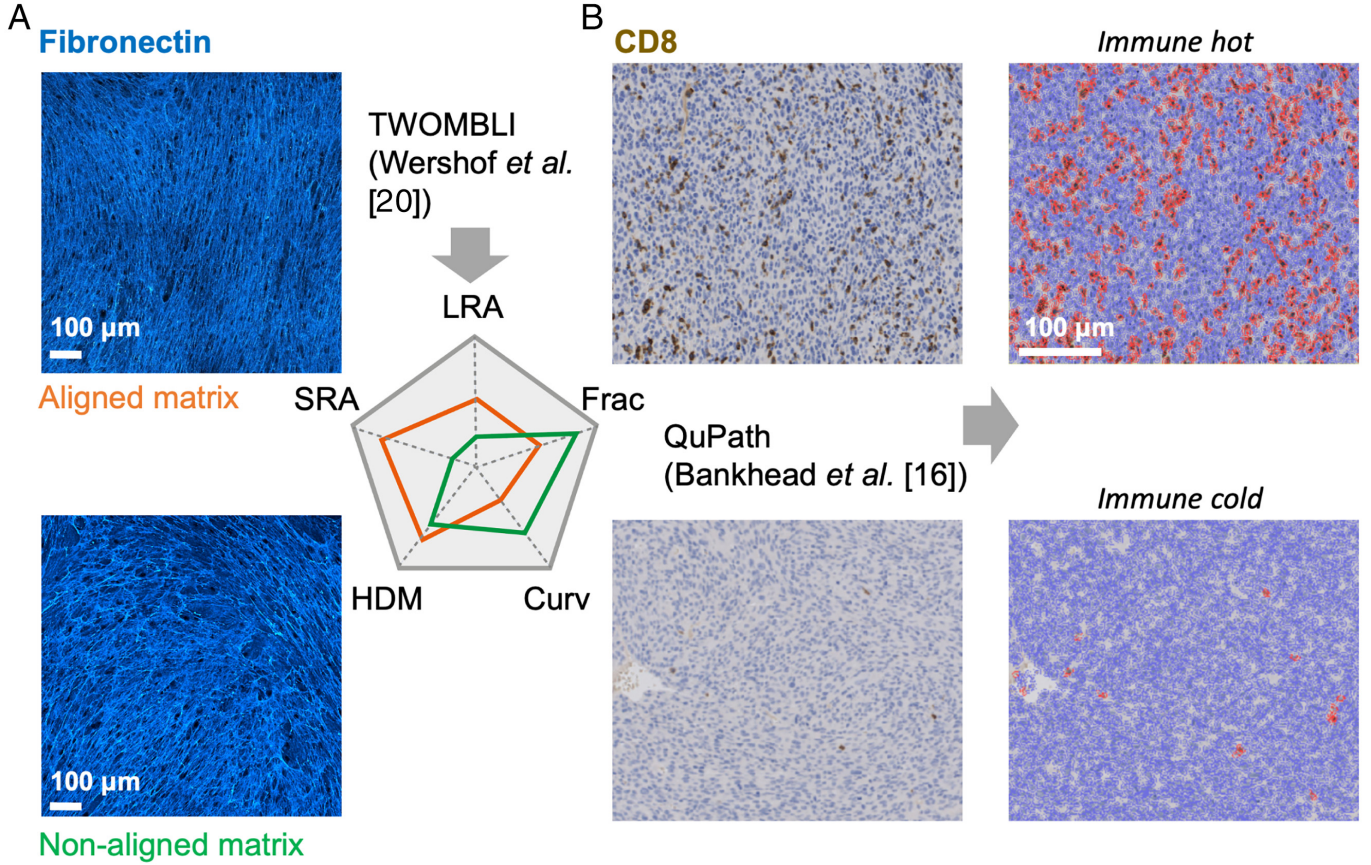
Exemplar analyses of quantitative feature extraction. (A) Quantitative matrix features extracted using TWOMBLI based on immunofluorescence for fibronectin. (B) Density of T cells extracted using QuPath based on CD8 staining. Curv, curvature; Frac, fractal dimension; HDM, high‐density matrix; LRA, long‐range alignment; SRA, short‐range alignment.

There is a wide range of image types to which AI algorithms are increasingly being applied. These include H&E‐stained sections – a simple and inexpensive part of standard diagnostic procedures that is widely available in routine pathology laboratories worldwide but lacks unambiguous cell type information. Considerable progress has been made in AI algorithms using H&E images, as discussed earlier [11, 14, 15]. Here, computational extraction of cell and nuclear size, as well as their shape, is fairly straightforward and precise identification of tumour cells versus fibroblast versus leukocyte is increasingly possible, e.g. using digital pathology image analysis tools such as QuPath [16] or convolutional neural networks (CNNs) [17]. The haematoxylin stain also enables the detection of specific chromatin structures [18, 19]. Furthermore, it is increasingly possible to link H&E images with the genome using AI algorithms, as discussed later. Immunohistochemical stains, e.g. for CD8+ T cells or cytokeratins for tumour cells, can be helpful in validating cell detection algorithms using H&E, as well as being useful in their own right for quantitative analysis. Non‐cellular components of the TME can be visualised using chemical stains such as Picrosirius red, Masson's trichrome or Gomori trichrome, which specifically detect the collagen ECM or immunofluorescence for other ECM components, such as fibronectin, as exemplified in Figure 1. Both immunohistochemistry and collagen stains are simple and widely available but are limited in that they characterise very few (typically one or two) components of the TME in each section.

With the recognition that the TME has a profound impact on tumour phenotype, construction of tissue atlases encompassing a range of cell types at subcellular resolution is increasingly important. Highly multiplexed platforms enable the detection of over 100 antigens on a single tissue section, with the obvious advantage of capturing the spatial complexity of the TME with much greater precision. Important multiplex platforms include imaging mass cytometry (IMC), Phenocycler (formerly CODEX), Akoya Biosciences (Marlborough, MA, USA), and VECTRA Polaris, Akoya Biosciences (Table 1). Of clinical relevance, formalin‐fixed paraffin‐embedded tissue can be used for all three platforms. IMC consists of high‐resolution (1 μm^2^) laser ablation and cytometry by time of flight to detect up to 40 antigens labelled with antibodies conjugated to metal tags [32]. Multiplexed ion beam imaging time of flight (MIBI‐TOF) is technically very similar to IMC, including the number of targets that can be visualised, but uses a tuneable ion beam that can be adjusted for tissue depth instead of a laser for tissue ablation [33]. Phenocycler uses fluorescent oligonucleotide‐based tagging of antibodies, which are sequentially hybridised and dehybridised across multiple cycles; automated microscopy is able to detect over 100 targets. In VECTRA Polaris, a secondary antibody is fused to a fluorescent opal dye and using up to six serial antigen retrieval cycles, six different targets plus a counterstain can be visualised on a single section [25]. Across platforms, there is a trade‐off between the number of targets visualised and the quantity of tissue that can practically be imaged. As a result, whole‐slide imaging using ultra highly multiplexed systems is considerably slower than with VECTRA Polaris; this is of practical relevance for discovery science and a very important consideration for the clinic, where inexpensive high throughput staining and scanning is essential.

**Table 1:** Types of image used in digital pathology.

Type of image	Brief description	Number of detectable targets	Examples
H&E	Routinely used stain in which haematoxylin precisely stains nuclear components, including heterochromatin and nucleoli, whereas eosin stains cytoplasmic components, including collagen and elastic fibres, muscle fibres, and red blood cells.	Unspecified	[10, 11, 14]
Picrosirius red staining	A widely used histological technique to visualise the distribution of collagen. The stain highlights the natural birefringence of collagen fibres when exposed to polarised light, enabling a detailed study of collagen organisation.	1	[20, 21]
Immunohistochemistry	A commonly used test in which an antibody detects a specific antigen or marker in a sample of tissue. The antibody is typically linked to an enzyme or a fluorescent dye, which is activated to enable visualisation using a microscope or digital scanner.	1–2	[22, 23, 24]
VECTRA Polaris	A secondary antibody is fused to a fluorescent opal dye and using up to six serial antigen retrieval cycles, six different targets plus a counterstain can be visualised on a single section	Up to 6	[25, 26]
IMC	High‐resolution (1 μm^2^) laser ablation and cytometry by time of flight is combined to detect up to 40 antigens labelled with antibodies conjugated to metal tags	Up to 40	[27, 28, 29]
Phenocycler (formerly CODEX)	Uses oligonucleotide‐based tagging of antibodies, which are sequentially hybridised and dehybridised across multiple cycles	Over 100	[30, 31]

**Figure 2:**
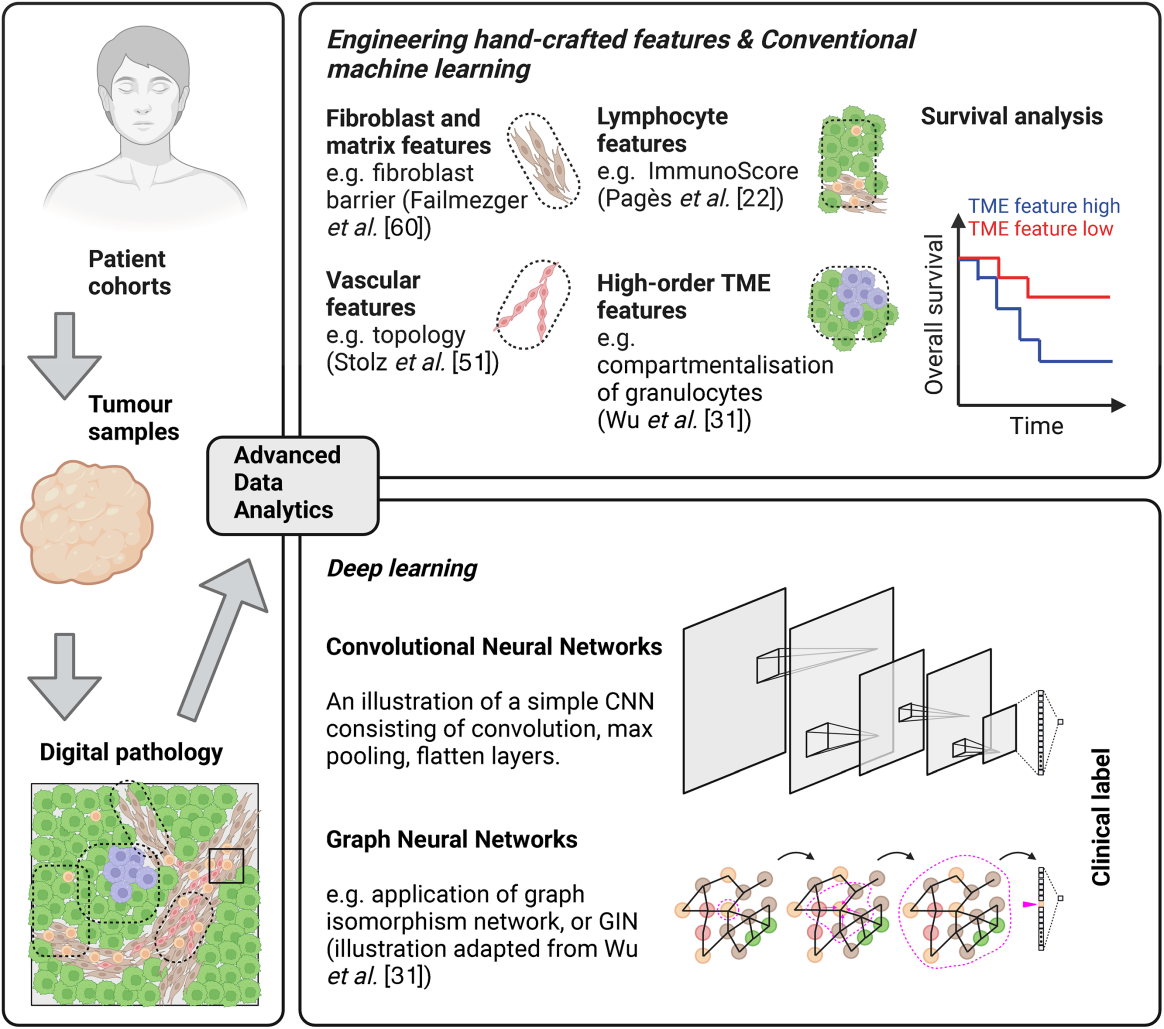
Prediction of clinical outcomes via conventional versus deep learning. Created with BioRender.com.

Concomitant with the advances in imaging techniques, innovative imaging data analysis and machine‐learning algorithms have been developed in the last couple of decades to assist in tumour diagnosis and understanding of clinically relevant tumour and microenvironmental features [1, 12, 34] (Figure 2). In the following sections we discuss recent progress and achievements in this area, with a focus on hand‐crafted feature engineering and deep‐learning studies that discovered clinically relevant TME features.

### What quantitative approaches are possible using digital images?

A range of machine‐learning models are used in the quantitative analysis of digital images. These range from less complex regression models to considerably more complex AI approaches. AI can play a role at different stages of the process of linking images to clinical parameters. First, AI can be trained to recognise features in images and extract metrics of those features. Second, features can be extracted using ‘rules‐based’ algorithms and AI then used to relate the output metrics to clinically important features/outcomes as conventional machine learning. Finally, AI can be used to perform both steps together, i.e. there is no need for prior feature extraction, this is referred to as deep learning.

Machine‐learning applications can be categorised as supervised, weakly supervised, and unsupervised, depending on the extent and type of data annotation [35]. Whereas in a supervised method the label needs to be provided for every data point (e.g. pixel‐level annotation of tumour versus normal tissue), in a weakly supervised method the label is given at the patient level (e.g. the whole‐slide H&E image contains areas of tumour). When the aim is to identify distinct phenotypes present in the data without labels, unsupervised methods, such as dimensionality reduction and clustering analysis, are applied.

In conventional machine learning, an image processing step prior to feature engineering is commonly segmentation to identify cell positions and classify cell types. Neural network models have been increasingly employed to perform image segmentation [36, 37, 38]. Subsequently, a set of quantitative features are extracted from a digital image based on certain mathematical descriptions, which are usually referred to as ‘hand‐crafted’ features (Figure 1). These are then applied to a conventional machine‐learning system, which establishes relationships between these input features and an output label, such as tumour diagnosis. In contrast, in deep‐learning AI approaches, the raw image is fed into the AI model, such as a CNN, which then progressively ‘learns’ which aspects of the image are most relevant for the outcome of relevance [1]. Both conventional and deep‐learning models usually require images to be broken down into a number of equally sized ‘tiles’ for computational tractability. In addition, each image needs to be labelled for the outcome of interest, i.e. cancer or no cancer, recurrence versus non‐recurrence, and this label will then be applied to all tiles in the image. As it is either laborious (cancer or no cancer) or infeasible (recurrence status) for pathologists to provide pixel‐level annotations for all images, weakly supervised learning that labels tiles according to slide‐level annotation has been commonly applied (e.g. [11]). The subsequent sections will discuss conventional machine‐learning and deep‐learning approaches to study the TME in greater depth.

## Conventional machine learning to map from hand‐crafted TME features to clinical outcomes

Quantitative hand‐crafted TME features are frequently employed to predict patient outcomes via the use of conventional machine learning. These features are commonly defined according to mathematical descriptions of the density, spatial distribution, and higher‐order structures of cancer cells and microenvironmental components. Conventional machine‐learning approaches, such as logistic regression and random forests, and survival statistical models, such as Cox proportional hazards model, are applied to map from features to clinical labels such as patient survival outcomes.

### Lymphocyte features

Evasion of immunosurveillance is a hallmark of many cancers and can arise from a deficiency of anti‐cancer immune cells (e.g. CD8+ cytotoxic T lymphocytes) or the presence of immunosuppressive elements (e.g. regulatory T cells) [39]. Consequently, features of immune contexture that represent the density, composition, and spatial organisation of immune components are shown to be correlated with cancer prognosis [40, 41, 42].

Imaging‐based quantitative analysis of immune contexture has led to the identification of immune features associated with patient outcomes. An international study involving 13 countries demonstrated that Immunoscore, which quantifies the density of CD8+ and CD3+ cells in tumour core versus invasive margin based on immunohistochemistry images, was a reproducible and robust prognostic factor in patients with stage I–III colon cancer [22]. Immunoscore was also shown to predict responses to chimeric antigen receptor T cell therapy in large B cell lymphoma [23]. A similar quantitative metric applied to more immune cell types based on immunohistochemistry images showed that immune topographies involving both CD8 and CD163 were prognostic [24].

Analysis of tumour‐infiltrating lymphocytes (TIL) has increasingly been facilitated by the application of CNNs, a type of deep learning that will be discussed in greater detail later. One CNN approach predicts the over‐ or under‐representation of TILs within individual small patches of a whole‐slide H&E image. Using this approach in a pan‐cancer analysis revealed spatial clustering features of TILs that associate with overall survival in some tumour types, including breast cancer and melanoma, and identified variable enrichment of structural patterns across tumour types [15]. Another approach seeks to detect individual cell nuclei and classify cell types within a whole‐slide H&E image. One such method is Spatially Constrained Convolutional Neural Network (SCCNN) [38]. This was used to reveal that intra‐tumour heterogeneity in immune landscapes has prognostic value. Facilitated by a machine‐learning pipeline for single‐cell identification and classification in H&E images, quantified geospatial features of lymphocytes in multiple tumour regions showed that lung adenocarcinomas with more than one region characterised by low lymphocyte infiltration had worse prognosis [17]. As discussed earlier, and enabled by multiplex imaging such as IMC and CODEX, higher‐order spatial immune cell neighbourhoods and interactions have been found to associate with survival outcomes and therapy outcomes in various tumour types, including cutaneous T cell lymphomas [43], melanoma [44, 45], colorectal cancer [46], and brain tumours [47].

Concomitant with the discovery of clinically relevant features based on digital pathology, experimental evidence increasingly sheds light on how immune contexture emerges from complex and dynamic immune cell behaviours. Combining immunostaining and dynamic imaging of T cells showed that stromal ECM density and orientation impacted T cell migration and localisation [48].

### Vascular features

Vascular systems play a fundamental role in the distribution of oxygen and nutrients to sustain tumour growth, as well as in the delivery of therapies. Disorganised tumour vasculature is a hallmark of cancer [39]. Microvessel density and fractal dimension, which measure the complexity of microvessel networks, extracted from CD34 immunochemistry images, were associated with survival outcomes in clear cell renal cell carcinomas [49]. More recently, quantitative morphometric features based on CD31 immunohistochemistry identified vascular features, including endothelial density and vascular arm numbers, associated with disease‐free survival in patients with clear cell renal cell carcinomas [50]. In addition to these quantitative metrics, topological data analysis, a set of methods to distil low‐dimensional features from high‐dimensional data, is emerging as a powerful mathematical tool for characterising vascular patterns. Multiscale topological descriptors of vascular patterns based on intravital images of mouse colorectal cancer quantitatively captured the dynamic changes in network architecture following anti‐cancer therapies [51].

### Fibroblast and matrix features

Fibroblasts plays a variety of important roles within the TME, including deposition and organisation of ECM and complex interactions with cancer cells and different types of immune cell [52]. Imaging‐based quantitative analysis of the organisation of stromal fibroblasts and matrix has led to the identification of features associated with patient outcomes. By overlaying second harmonic generation (SHG) images of collagen with H&E images, Conklin *et al* [53] devised a metric called tumour‐associated collagen signature‐3 (TACS‐3) to describe a pattern in which bundles of aligned collagen were oriented perpendicular to the tumour boundary in breast cancer. They found that a positive TACS‐3 score was correlated with unfavourable survival outcomes using a Cox proportional hazards model. Beck *et al* [54] extracted a rich set of quantitative features of epithelial and stromal compartments in breast cancer and discovered that stroma morphological features and their contextual relationships with cancer cells had prognostic value for patient survival. Yuan *et al* [14] reported that a quantitative score characterising the degree of spatial clustering in contrast to the randomly scattered distribution of stromal cells based on H&E‐stained images was associated with poor outcomes for breast cancer patients.

Various tools have been developed to facilitate the extraction of quantitative matrix features. A FIJI ImageJ plugin called TWOMBLI was developed to extract a diverse repertoire of quantitative metrics from Picrosirius red or SHG images, including the number of end points and branch points, high‐density matrix, curvature, alignment, and fractional dimension [20]. These metrics have shown clinical relevance, e.g. ECM alignment in the metastatic potential of prostate tumours [55] and collagen density and curvature in bladder cancer progression [56]. The TWOMBLI tool [20] can also be applied to extract features of blood vessels. A fibre segmentation and extraction MATLAB tool was developed that enabled quantitative analysis of collagen architectural features in SHG images [21]. In addition to fibre‐level analysis, quantitative features derived from gaps between fibres were shown to contain biologically relevant architectural information [57]. Recently, a machine‐learning approach was developed to construct SHG‐like representation of collagen directly based on H&E images, which enabled non‐destructive extraction of quantitative matrix features such as fibre orientation and alignment [58]. Overall, in contrast to lymphocyte features, quantitative features of vascular or matrix patterns are less developed and a consensual quantitative metric that predicts clinical outcome has not yet emerged.

### High‐order TME features

An increasing body of work also sought to extract higher‐order features from H&E‐stained images that describe the composition and spatial organisation involving multiple TME components [14, 59, 60]. Enabled by an automated computational pipeline to segment and classify cell nuclei, a set of features was engineered that describe the nuclear morphologies of cancer cells, stromal cells, and lymphocytes, demonstrating that integration of these histological features with genomic features improved the prediction of survival of oestrogen receptor‐negative breast cancer patients [14]. An ecological diversity index to quantitatively characterise the spatial variation in the local composition of cancer cells, stromal cells, and lymphocytes revealed that high microenvironmental heterogeneity was linked with worse disease‐free survival in breast cancer patients [59]. Characterising patterns of spatial cell–cell networks using a graph‐based approach found that a high extent of both stromal clustering and barrier appeared to suppress lymphocyte infiltration into tumours and was associated with poor survival outcomes in melanoma patients [60]. A set of cell‐level and tissue‐level quantitative features of cancer cells and four TME cell types identified from H&E images of multiple tumour types indicated that these human‐interpretable features were able to predict clinically relevant molecular phenotypes, such as PD‐1 expression [61].

With the recent advances of spatially resolved multiplex imaging techniques, such as VECTRA Polaris, MIBI‐TOF, IMC, and Phenocycler (CODEX), an increasing body of research has identified clinically relevant higher‐order quantitative features characteristic of spatial cell communities and organisation of TMEs [27, 28, 30, 62]. Quantitative characterisation of co‐occurrence, interactions, and spatial enrichment of immune cell populations in MIBI‐TOF images of triple‐negative breast cancer revealed that spatially mixed, in contrast to compartmentalised, tumour‐immune organisations were associated with poor survival outcomes [62]. Using IMC images of breast cancer, quantitative characterisation of pairwise cell neighbourhoods and higher‐order cell communities showed that spatial multicellular features had superior predictive power of overall survival in comparison to clinically defined subtypes [27]. In another study, cell population composition transitioned at tissue interfaces in IMC images of breast cancer and higher‐order multicellular structures were associated with genomic features and predictive of clinical outcomes [28]. Using CODEX images of advanced‐stage colorectal cancer, comprehensive analysis of the organisation, functional state, and communication patterns of cell neighbourhoods uncovered spatially resolved multicellular features associated with effective antitumour immunity and survival outcomes [30].

The future development of spatially resolved imaging, such as 3D methods [63] and spatial high‐plex profiling of RNA and protein expression [64], combined with advanced data analysis [65, 66], is set to further provide a refined depiction and understanding of the TME. As hand‐crafted feature engineering and machine learning increasingly show utility in the discovery of TME patterns associated with clinical outcomes, there remains a largely unmet need – a promising opportunity for understanding better the mechanistic underpinning of such patterns in pre‐clinical laboratory research.

## Deep learning to predict clinical outcomes and beyond

Deep‐learning approaches have emerged as a powerful tool in digital pathology applications, such as tumour diagnosis and the inference of genotypes, without the need to explicitly engineer hand‐crafted features. These approaches commonly employ algorithms capable of discovering relevant contextual patterns inherent in the input data and often require only a minimum level of human intelligence as input. Deep‐learning approaches, therefore, have the potential to achieve both clinically relevant predictions and discovery of important features, which can enable further biological and experimental hypothesis generation. The development of deep‐learning biomedical applications requires a large amount of data with clinical annotations and has benefited from pan‐cancer, multimodality datasets curated in landmark programmes such as The Cancer Genome Atlas (TCGA). In the following sections, we discuss a variety of digital pathology applications using deep learning.

### Convolutional neural networks

CNNs are the most widely applied deep‐learning method in digital histopathology applications (Table 2). Digital pathology applications of CNNs, and deep learning in general, benefit from the success of transfer learning, namely reuse of neural network models extensively trained for image classification problems with abundant training data, without the need to retrain all layers. CNNs have been extensively used to predict patient outcomes in recent years and demonstrate model performance that is comparable or superior to human experts, as discussed earlier and below. Despite the ‘black‐box’ nature, researchers have developed methods to visualise domains within images associated with model prediction and therefore inform the clinically relevant tumour and microenvironmental features.

**Table 2:** Types of deep‐learning model.

Type of model	Brief description	Examples
CNNs	In its core part, a CNN applies a mathematical operation called a convolution to pixel intensities within an input image and is hierarchically structured with layers of operations to represent features at varying scales within the image. Variants of the basic CNN, including Inception‐V3 and ResNet, are among the best models in digital pathology applications.	[11, 29, 67, 68, 69, 70, 71, 72, 73]
AMs	An important feature of AMs, compared to CNNs, is the explicit representation of a non‐uniform contribution of information in different parts of the input data as a trainable property of the neural network. Therefore, AMs make biological interpretation convenient, e.g. by outputting the relative importance of subdomains within the input image(s) for predicting patient outcomes.	[74, 75, 76]
GNNs	GNNs work on graphs constructed based on pre‐processed biological landmarks, such as different types of cell, in contrast to pixel intensities. Therefore, GNNs can explicitly build in the structure of multicellular communities and cell–cell communications.	[31, 77]
MMs	MMs integrates, as streams of model input, multiple modalities of data, such as digital pathology images, genomic sequencing data and clinical annotations, such as tumour grade. MMs can enable the assessment of the relative contribution of input modalities to predictions.	[78, 79, 80]

Campanella *et al* [11] developed a deep‐learning framework that performed well without pixel‐level annotation of tumour areas in the diagnosis of prostate cancer, basal cell carcinoma, and breast cancer based on whole‐slide H&E images. Courtiol *et al* [67] developed a CNN model predicting patient outcomes based on whole‐slide H&E images of mesothelioma and found that histological stromal features were associated with poor survival outcomes. Kather *et al* [68] trained CNN models to form internal representations of different tissue classes based on histological images of colorectal cancer and showed that a ‘deep stroma score’ related to the representation of the stromal compartment was associated with survival outcomes.

In addition to the classification of clinical outcomes, multiple studies have shown that CNNs are also capable of predicting genomic alternations in various cancer contexts. A CNN model was able to classify subtypes of non‐small cell lung cancer (NSCLC) and predict the mutational status of six of 10 most frequently mutated genes from histological images [69], although as discussed later, not with the precision seen using next‐generation sequencing. CNN models were also able to predict the status of microsatellite instability in colorectal cancer based on H&E images [70]. Two concomitantly published studies by Kather *et al* [71] and Fu *et al* [72] showed that CNN‐based deep‐learning algorithms could predict diverse molecular alterations, including gene mutations and transcriptional profiles, directly from histology in a pan‐cancer context. Both studies also found an association of TME features with CNN prediction of genomic alterations. Kather *et al* [71] also reported that the enrichment of stroma was associated with CNN prediction of consensus molecular subtype 4 (CMS4) in colorectal cancers. Another CNN model accurately classified CMS based on H&E images of rectal cancer [81]. Image‐based CMS, associated the predictions of molecularly determined classes with histological features of TME organisation, such as lymphocytic infiltrates and desmoplastic stromal reaction, and demonstrated value in investigating intratumoural transcriptional heterogeneity. Overall, it remains unclear in these studies to what extent TME features were associated with CNN predictions of genomic alterations. Although these methods innovatively demonstrated the feasibility of predicting genomic alternations based on digital pathology images, the current precision is inferior to detection using next‐generation sequencing, as discussed later. Further improvements are required to enable clinical translation.

Recent studies also sought to apply CNN models to image data beyond H&E slides, concomitant with the advances of spatially resolved imaging. By combining matched H&E staining and spatial transcriptomics data for model training, He *et al* [73] developed a CNN‐based algorithm to predict expression profiles of 250 genes in a proof‐of‐concept study based on a dataset of 23 patients with breast cancer. Using IMC images as training data, Sorin *et al* [29] developed a CNN‐based framework that processes and integrates information of individual marker stains of IMC images – this is outlined in detail later but an important finding was that a combination of five markers could predict survival outcomes of patients with NSCLC.

### Attention‐based models

Attention‐based models (AMs) have transformed other machine‐learning fields, such as language translation, and, recently, have started to have footprints in digital histopathology applications to cancer research (Table 2). AMs achieved good performance in the subtyping of renal cell carcinomas and NSCLC and were able to highlight characteristic morphological features on the whole‐slide images [74]. In a follow‐up study, an AM‐based framework was developed that could simultaneously predict whether a tumour is a primary or metastasis and the site of its origin across multiple tumour types [75]. More recently, an AM was developed that predicted prognosis and therapy response in colorectal cancer based on immunohistochemistry images of four immune cell markers and explained the relative importance of markers via the built‐in attention module [76].

### Graph neural networks

Graph neural networks (GNNs) have been widely applied in other biological research fields, such as protein structure predictions [82]. Recent methodology developments led to promising applications to digital histopathology images in cancer research (Table 2). In application to imaging data, GNNs are commonly applied to graphs constructed based on pre‐processed biological landmarks, such as different types of cells, in contrast to pixel intensities.

Applying a GNN to a spatial graph connecting segmented cells from H&E images of tissue microarrays of prostate cancer showed that the algorithm was able to classify Gleason scores [77]. A GNN based on CODEX images of head and neck and colorectal cancers demonstrated that the model was able to predict survival outcomes and identify disease‐relevant cellular communities, such as compartmentalisation of granulocytes [31].

### Multimodal models

In addition to approaches based on digital histopathology images alone, multimodal models (MMs) that integrate multiple modalities of data as streams of model input are emerging to become an exciting avenue of deep‐learning application. Mobadersany *et al* [78] developed a MM framework integrating both histopathology images and genomic markers and achieved superior prediction of overall survival of glioma patients. They further showed that the model linked higher risk with histological features, including microvascular proliferation and high tumour cell density. Esteva *et al* [79] developed a MM based on both histopathology images and clinical variables such as Gleason score and tumour stage and showed that the model improved prognostication of patients with prostate cancer in randomised clinical trials with long‐term follow‐up. In a recent pan‐cancer study, Chen *et al* [80] developed a multimodal AM that integrates digital histopathology and molecular profile data to predict patient outcomes across 14 cancer types. Their approach led to the identification of prognostic morphological and molecular features correlated with outcomes. They also found that their MM attributed attention to areas of tumour‐associated stroma in high‐risk cases of pancreatic adenocarcinoma, suggesting a role for TME features in the model prediction of survival outcomes.

### Other deep‐learning approaches

A variety of exciting deep‐learning applications have focused on other biologically and clinically important research areas, including real‐time AI to assist in intraoperative diagnosis [83, 84, 85], biologically inspired AI to improve interpretability [86], efficient search of archival histopathology images to facilitate decision‐making [87, 88] and federated learning to encourage cross‐centre collaboration and protect data privacy [89].

## Challenges for the implementation of digital image AI – clinical and methodological

### What can digital pathology advanced analytics achieve in the clinic?

An important clinical question is: what can AI algorithms as biomarkers realistically achieve to improve patient care? Biomarkers are typically thought of as diagnostic, prognostic or predictive of response to any specific therapy. Predictive biomarkers are particularly useful to direct personalised therapy choices but to date have been challenging to develop robustly and are infrequently available.

### Progress in the use of AI in diagnostics and prognostication

In terms of diagnostic AI algorithms it is clear that in specific tumours, e.g. prostate cancer, there has been considerable progress in diagnosis, some of which are now available for diagnostic clinical use [10, 11]. Cancers of unknown primary are tumours where it is difficult to define the primary site of origin. Tumour Origin Assessment via Deep Learning (TOAD) is a recently published high‐throughput interpretable deep learning‐based solution that uses H&E whole‐slide images to predict whether a tumour is primary or metastatic and to ascribe a differential diagnosis for the primary site of origin [75]. Transfer learning and weakly supervised multitask learning were combined to train a unified predictive model. In addition, attention‐based learning located slide regions of particular diagnostic relevance, which were validated by pathologists. On an external test cohort of cancer of unknown primary, TOAD showed an accuracy of 79.9% in diagnosing a primary site of origin. For distinguishing between a metastasis and a primary tumour, the model had an impressive area under the curve (AUC) of 0.919 in the external test cohort. TOAD illustrates how AI algorithms using inexpensive routinely available diagnostic tissue can help pathologists with complex diagnostic decisions and reduce diagnostic workup times.

In terms of prognostic biomarkers, once again AI algorithms based on digital AI images have already shown impressive results. The Gleason score in prostate cancer is a measure of glandular differentiation and a well‐established prognostic factor that is subject to inter‐pathologist variation in scoring [90]. In recent years, several AI algorithms have been described using H&E images that attribute the Gleason score with an accuracy equivalent to that of specialist pathologists [91, 92]. A further important growth in AI approaches to prognosis has been in extracting clinically relevant spatial relationships between different TME features from highly multiplex immunofluorescence images. Sorin *et al* [29] recently used IMC with 35 markers on 1 mm tissue microarray cores and a pre‐trained neural network model that combined routine clinical parameters plus spatial cell information, including measures of cellular TME communities, to predict recurrence following lung cancer surgery. Intriguingly, the deep‐learning model substantially improved in predictive ability with the specific incorporation of spatial information, but not with just cell frequencies, and achieved 95.9% accuracy in predicting recurrence in stage I NSCLC. The size of tissue used in this study corresponds well to the small diagnostic biopsies available in the clinic. Furthermore, the authors were able to reduce the number of markers and still achieve a predictive accuracy of 90.8% using CD14, CD16, CD94, αSMA, and CD117, suggesting that more clinically practical lower plex methods involving important TME targets add clinical benefit.

Despite the promise of AI algorithms in diagnosis and prognostication, potential risks of widespread incorporation into clinical practice include the deskilling of pathologists, e.g. in the detection of less common tumour patterns. Furthermore, it is not yet clear how well digital pathology‐based AI algorithms perform in the detection of atypical tumour variants, e.g. prostate sarcoma rather than adenocarcinoma. In specific cases, pathologists will decide to section deeper and perform additional tests in the face of diagnostic uncertainty; it is possible that AI algorithms are unable to recommend the optimal management of such ambiguous cases.

### Limitations in the use of AI for therapy selection

AI algorithms based on digital images to predict benefit from a specific therapy have not yet achieved the same accuracy as the previously described diagnostic and prognostic AI biomarkers. This is partly because detection of a specific genetic aberration is often required from digital images. Here, AI models provide little added value if the key information can be obtained by sequencing, except potentially reducing cost in some contexts. Using CNNs based on modified inception v3 architecture and whole‐slide images of H&E‐stained NSCLC from the TCGA lung cohort, the authors trained a model to identify 10 commonly mutated genes in adenocarcinoma of the lung, which they compared with next‐generation sequencing data [69]. The resulting AUC values for the detection of mutations in serine/threonine protein kinase 11 (*STK11*), *EGFR*, FAT atypical cadherin 1 (*FAT1*), SET binding protein 1 (*SETBP1*), *KRAS*, and *TP53* were between 0.733 and 0.856. Testing these predictive models in an independent dataset showed an AUC of 0.687 (CI 0.554–0.811), with a higher AUC (0.75; CI 0.500–0.966) in samples validated by sequencing than in those tested by immunohistochemistry (AUC 0.659; CI 0.485–0.826). These AUC values are similar to those seen in prostate cancer, where a deep learning‐based predictive model was able to identify SPOP mutations with an AUC of 0.71 [93]. The above results are important, not least because KRAS and EGFR have specific drug candidates in the clinic. At present the accuracy of these models is not sufficient to guide the choice of therapy, but emerging larger datasets will improve model training and potentially reach a precision that is acceptable for clinical use. As discussed later, the deep‐learning AI methodology used in these studies means it is difficult to ascertain how much the TME informs the eventual decision regarding mutation status.

In other personalised therapy contexts, there is no gene mutation to guide treatment selection but better predictive biomarkers are still urgently needed. The selection of patients who might benefit from immunotherapy is a relevant example where TME constituents play an important role in driving the response or resistance to the immunotherapy. A second example is the prediction of which patients with localised prostate cancer need additional androgen deprivation therapy alongside radical radiotherapy. Encouragingly, an AI‐based marker has recently shown promise in filling this gap. Using self‐supervised learning, a Resnet‐50 feature extraction model was trained on image patches from H&E images of prostate biopsies from 5,000 patients recruited to five phase III randomised trials of radiotherapy plus or minus androgen deprivation therapy [94]. When applied to the validation or test set of 1,594 patients, there was a significantly positive biomarker treatment test for interaction (p‐interaction = 0.01). These results require further validation, and it is relevant that the size of the datasets were small compared with AI studies outside of oncology, e.g. those used in animal recognition. Nonetheless, the findings are exciting because the test is the first predictive marker generated in this context. In addition, H&E images are practically easier and less expensive to acquire than the gene expression signatures currently used as prognostic biomarkers in localised prostate cancer [95].

### Linking digital pathology‐based advanced analytics with clinical imaging modalities

A further largely unexplored research opportunity with the potential to enhance clinical decision‐making is linking digital pathology‐based advanced analytics with routinely used clinical imaging modalities, such as ultrasound, CT, and MRI. AI approaches using CNNs to link radiomics data from CT and MRI with clinical outcomes, e.g. in the diagnosis of liver metastasis from colorectal tumours, have recently expanded [96, 97]. Chen *et al* [98] demonstrated the utility of CNN in predicting the methylation status of the O^6^‐methylguanine methyltransferase (MGMT) gene promoter in glioblastoma multiforme, a prognostic biomarker, based on fluid‐attenuated inversion recovery (FLAIR) MRI images. Furthermore, the relationship between digital pathology‐based TME features, such as collagen morphology and mammographic features, is well established [99]. Beyond this, there is considerable scope to link more complex digital imaging features with widely available clinical images. AI algorithms have the computational capacity to overcome the challenges in combining such diverse data types using MMs. In addition, clinical images often visualise an entire tumour longitudinally, which enables detailed mapping of temporal and spatial heterogeneity in the TME.

### Challenges of validation and qualification

Robust statistical validation and qualification is essential for novel AI algorithms based on digital pathology to translate to the clinic. The Reporting recommendations for Tumour MARKer prognostic studies (REMARK) guidelines were developed following the observation that ‘…despite years of research and hundreds of reports on tumour biomarkers in oncology, the number of markers that have emerged as clinically useful is pitifully small…’ [100, 101, 102]. The REMARK recommendations include adjustment for multiple testing and use of adequate measures of discrimination and calibration of any novel biomarker, which is particularly relevant in predicting prognosis. Both AUC and the concordance index (c‐index) are useful measures of discrimination and an important judge of clinical utility is the improvement in AUC or c‐index seen with the addition of the novel biomarker to standard clinical factors versus such factors alone [103, 104].

Previous reporting guidelines including Standard Protocol Items: Recommendations for Interventional Trials (SPIRIT) [105] and Consolidated Standards of Reporting Trials (CONSORT) [106] are not readily applicable to clinical trials based on AI systems. Recently, SPIRIT‐AI [107, 108, 109] and CONSORT‐AI [110, 111, 112] have been developed to provide standards for designing and conducting clinical trials based on AI systems [113]. These guidelines provide AI‐specific items in addition to the core items described in SPIRIT and CONSORT. Alternative guidelines are currently being developed [114, 115]. Independently, SPIRIT‐Path is another guideline extending from SPIRIT to recommend items on reporting of cellular and molecular pathology content [116]. With the proposal of multiple guidelines for the implementation of AI‐facilitated clinical trials, a challenge emerges in the choice of which guideline to apply in specific clinical settings. A further challenge is how to enable the ongoing training and thus improvement in AI algorithms versus needing to ‘lock’ an AI algorithm at a defined point in time prior to approvals for clinical use.

The explainability of AI applications is an important consideration for clinical use and there is ongoing debate about whether inherently interpretable machine‐learning models should be advocated more than black‐box models assisted by *post hoc* explanation [117, 118]. Some of the core discussion points in the debate include whether black‐box models necessarily have superior predictive performance and whether *post hoc* explanation of black‐box models, such as saliency maps in CNNs or attention maps in AMs, may be misleading or insufficient. Future AI applications will need to encourage attempts to develop biologically inspired interpretable machine‐learning models [86], as well as critically assessing the biological/clinical relevance of *post hoc* explanations of black‐box models.

A further challenge for the clinical implementation of digital pathology‐based AI algorithms is the need for prospective evaluation [119]. For predictive biomarkers, the gold standard is a prospective randomised controlled trial where patients are randomised according to the current standard of care or the novel biomarker score [119]. This expensive and lengthy prospective qualification is rarely carried out and, in reality, gene expression profiling signatures, e.g. Oncotype DX in breast cancer, have often entered routine clinical use following repeated retrospective validation and before prospective qualification is complete [120, 121]. Digital pathology‐based AI algorithms have almost entirely been evaluated retrospectively and, to our knowledge, have yet to be reported in a prospective setting. Unless this is addressed, there is the danger of a growing misfit between AI methodology and the well‐established rigour of clinical trials. Equally, traditional randomised controlled trial methodology is not always suitable for the evaluation of AI technology; addressing a potential misfit requires clinical trialists to identify novel approaches to trial design that can accelerate approval of clinically beneficial digital pathology‐based AI algorithms.

Prostate cancer diagnosis in a prospective setting is being tested in an ongoing multidisciplinary study called ARTICULATE PRO by integrating pathologists' decisions with the use of Paige Prostate. Arguably, one critical element of successful AI implementations in prospective, in contrast to retrospective, settings is the human–AI interaction, as experts need to conduct live appraisal of recommendations by AI algorithms and make decisions on clinical practice. Guidelines like DECIDE‐AI [122, 123, 124] have been developed to provide standards for reporting on the evaluation of AI systems in live clinical settings of patient care.

## Summary and conclusions

The combination of highly multiplex platforms to profile the TME and increasing powerful computational models means there is exciting potential for enhanced clinical decision‐making using digital pathology‐based advanced analytical methods. In order to realise this potential, relevant studies need to incorporate meaningful measures of ‘added value’ to the current standard of care, incorporate prospective validation and meet the high standards of reliability and robustness set out in guidelines such as REMARK [100].

Engineering hand‐crafted features combined with conventional machine‐learning approaches has the advantage of delivering clear interpretability in terms of which key features correlate with clinical outcomes. However, these approaches are often less successful than deep‐learning methods, partly because the set of hand‐crafted features can under‐represent the complexity in digital histopathology. Therefore, there is a trade‐off between model performance and interpretability. Deep‐learning models have achieved good performance in the prediction of patient outcomes and demonstrated promising results in predicting genotypes from digital pathology images. However, there remains a need for interpretation, led by pathologists, of biological patterns within the input image captured by the model.

Of note, in both types of model framework and digital pathology application, interpretability remains largely limited from the perspective of mechanistic principles underpinning the formation of clinically relevant patterns. Whereas model interpretation sheds light on what kinds of TME organisation inform clinical outcomes, understanding why, and how, such patterns underlie disease progression will require pre‐clinical mechanistic laboratory research. Integration of experimental insights into the analysis of histopathology images during model development will be an exciting avenue for the future progress of AI applications.

## Author contributions statement

XF and AW drafted the original manuscript, tables and figures, ES provided advice on the structure and critical review of the manuscript. All authors agreed the final version to submit for publication.
